# HIV MADE ME STRONGER: CONCEPTIONS OF SUCCESSFUL AGING AMONG OLDER WOMEN LIVING WITH HIV

**DOI:** 10.1093/geroni/igz038.2812

**Published:** 2019-11-08

**Authors:** Anna A Rubtsova, Tonya Taylor, Gina Wingood, Igho Ofotokun, Deborah Gustafson, Marcia Holstad

**Affiliations:** 1 Emory University Rollins School of Public Health, Atlanta, Georgia, United States; 2 SUNY Downstate Medical Center, Brooklyn, New York, United States; 3 Mailman School of Public Health, Manhattan, New York, United States; 4 Emory University, Atlanta, Georgia, United States

## Abstract

Little is known about successful aging (SA) among older (50+) women living with HIV (OWLH). Therefore, the purpose of this qualitative study was to examine subjective understandings of SA among OWLH. Participants were OWLH enrolled in the Women’s Interagency HIV Study (WIHS) and those not enrolled in WIHS: 8 in Atlanta, GA (3 WIHS and 5 non-WIHS) and 9 in Brooklyn, NY (6 WIHS and 3 non-WIHS). Semi-structured interviews ranged from 30 to 120 minutes. Several themes emerged in participants’ definitions of SA, e.g. self-care, taking HIV medications, and being resilient (“HIV made me stronger”). Both WIHS and non-WIHS participants emphasized life course perspective in their definitions of SA -- women viewed their aging successful as a more stable phase of life in contrast to hardships they experienced while being younger (e.g., drug use, incarceration). Data collection efforts are ongoing and will allow further characterization of SA among this population.

